# Ecophysiological traits of highly mobile large marine predators inferred from nucleic acid derived indices

**DOI:** 10.1038/s41598-020-61769-7

**Published:** 2020-03-16

**Authors:** F. Alves, M. Dromby, V. Baptista, R. Ferreira, A. M. Correia, M. Weyn, R. Valente, E. Froufe, M. Rosso, I. Sousa-Pinto, A. Dinis, E. Dias, M. A. Teodósio

**Affiliations:** 1 MARE - Marine and Environmental Sciences Centre, ARDITI, Madeira, Portugal; 2 OOM - Oceanic Observatory of Madeira, Funchal, Portugal; 3 Faculty of Sciences and Technology, Universidade do Algarve, Campus de Gambelas, Faro, Portugal; 4 CCMAR - Centre of Marine Sciences, Universidade do Algarve, Campus de Gambelas, Faro, Portugal; 5 CIIMAR - Interdisciplinary Centre of Marine and Environmental Research, University of Porto, Terminal de Cruzeiros do Porto de Leixões, Matosinhos, Portugal; 6 Department of Biology, Faculty of Sciences, University of Porto - FCUP, Porto, Portugal; 7 Marine Biology Research Group, Ghent University, Ghent, Belgium; 8 CIMA Research Foundation, Savona, Italy

**Keywords:** Biological techniques, Chemical biology, Ecology

## Abstract

Nucleic acid-derived indices such as RNA/DNA ratios have been successfully applied as ecophysiological indicators to assess growth, nutritional condition and health status in marine organisms given that they provide a measure of tissue protein reserves, which is known to vary depending on changes in the environment. Yet, the use of these biochemical indices on highly mobile large predators is scarce. In this study, we tested the applicability of using nucleic acids to provide insights on the ecophysiological traits of two marine mammal species (common bottlenose dolphins and short-finned pilot whales) and explored potential related factors (species, sex, season, and residency pattern), using skin tissue (obtained from biopsy darts) of apparently healthy and adult free-ranging animals. Significantly higher RNA/DNA ratios were obtained for bottlenose dolphins (p < 0.001), and for visitor pilot whales when compared with resident pilot whales (p = 0.001). No significant changes were found between the sexes. Based on the percentile approach, the samples contain individuals in a general good condition (as the 10^th^ percentile is not closer to the mean than the 75^th^ percentile), suggesting that the studied region of Macaronesia may be considered an adequate habitat. The combination of this effective tool with genetic sexing and photographic-identification provided an overall picture of ecosystem health, and although with some limitations and still being a first approach, it has the applicability to be used in other top predators and ecosystems.

## Introduction

Understanding the physiology of an organism in function of its environment and the factors contributing to its variability can enable assessment of the relative impacts of anthropogenic and ecological pressures, which is essential in a changing world^1,2^. Among the most used ecophysiological indicators at the organism level in marine ecology are nucleic acid-derived indices, such as RNA/DNA ratios, RNA/mg and DNA/mg. Especially the former has been successfully applied as indicator of growth, nutritional condition and health status in marine organisms, as well as indicator of natural or anthropogenic impacts in marine populations and communities^3^. This is based on the fact that the concentration of cellular DNA is relatively constant in the somatic cells regardless of any changes in the organism’s environment, while the RNA content of a cell increases as the cellular demand for protein synthesis and growth increases^4,5^. It thus provides a measure of cellular protein synthesis capacity, which is generally interpreted as an indicator of tissue protein reserves, and that varies depending on changes in the environment where organisms live, such as food and habitat availability or physical factors^6–8^. Nevertheless, the use of these biochemical indices is unbalanced in favour to growth-related studies of microbial, invertebrate, fish and reptile communities^9–13^, in detriment to nutritional condition and health studies of highly mobile megafauna/apex predator species that may serve as potential bioindicators of the ecosystem.

Large-sized apex predators, such as cetaceans (marine mammals), play an important role in maintaining the structure and function of the environment they inhabit^14,15^, and are especially affected by increasing global anthropogenic pressures (e.g. direct and indirect catches, habitat destruction)^16–18^. Obtaining information into these species’ ecophysiological traits is thus important because of their conservation management. However, gathering physiological information from free-ranging cetaceans is challenging. Based on a commonly used technique of obtaining skin tissue at sea^19^, the present study tested the use of nucleic acid-derived indices to provide insights into the nutritional condition and health status of two cetacean species. To our best knowledge, this is the first study using RNA/DNA ratios as a bioindicator in cetaceans, and to a larger extent in wild mammals or highly mobile large predators.

The two cetacean species used in this study are the common bottlenose dolphin (*Tursiops truncatus*) and the short-finned pilot whale (*Globicephala macrorhynchus*). While the former is a mid-sized delphinid living in a fission-fusion society, is commonly found in coastal and oceanic temperate and tropical waters, and feeds on a large variety of bottom-dwellers and pelagic fishes and/or squids^20,21^, the latter is a large-sized delphinid living in a matrilineal society, occurs in oceanic habitats from tropical to warm-temperate waters, and feeds preferentially on squids caught during deep vertical dives^22,23^. On a global scale, both species have their conservation status described as least concern and their population trends are unknown^24,25^. In the north-east Atlantic, these species are considered common in the biogeographical region of Macaronesia^26^, which includes Madeira and the neighbouring archipelagos of the Azores and Canaries^27–29^. In this region, and for each species, animals with distinct residency patterns have been identified^30–33^, and genetic and photo-identification studies have shown inter-archipelago connectivity^34–37^. Yet, there is no information on these animals’ physiological condition to properly infer on their nutritional condition or health status, as neither on the influence of biological and environmental factors.

Taking advantage of the privileged location of Madeira to study the ecology of highly mobile large predators in a truly pelagic environment, our main goal was to test the applicability of using RNA/DNA ratios for assessing the ecophysiological condition in two cetacean species of different size and with distinct social structure and feeding habits, based on a multi-disciplinary approach. As specific goals, we explored (i) whether there are interspecific differences, and (ii) factors that may affect nutritional condition and health status at the intraspecific level, such as (a) sex, due to possible behavioural or hormonal differences, (b) season, due to possible environmental and/or gestation/lactating intra- and inter-annual differences, and (c) residency pattern, due to possible differences in the spatial structure and/or prey types.

## Material and methods

### Study site and data collection

**Figure 1:**
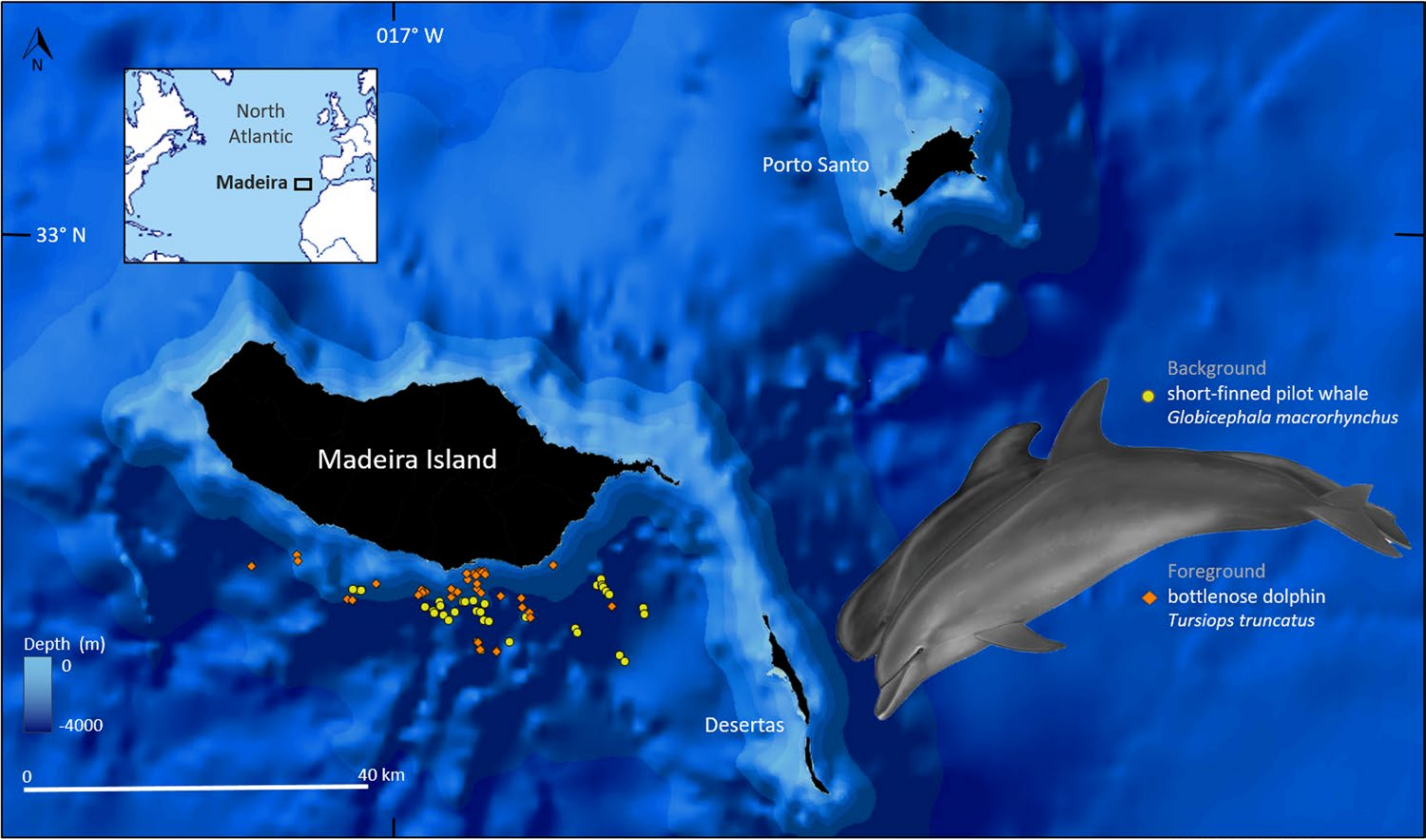
Location of Madeira and of the biopsied common bottlenose dolphins and short-finned pilot whales during 2017 and 2018 (map created with the software QGIS 2.18.15 https://www.qgis.org/en/site/). Species illustration by Les Gallagher - Fishpics & IMAR-DOP, UAç (the pilot whale is illustrated as a sub-adult in relation to an adult bottlenose dolphin).

**Figure 2:**
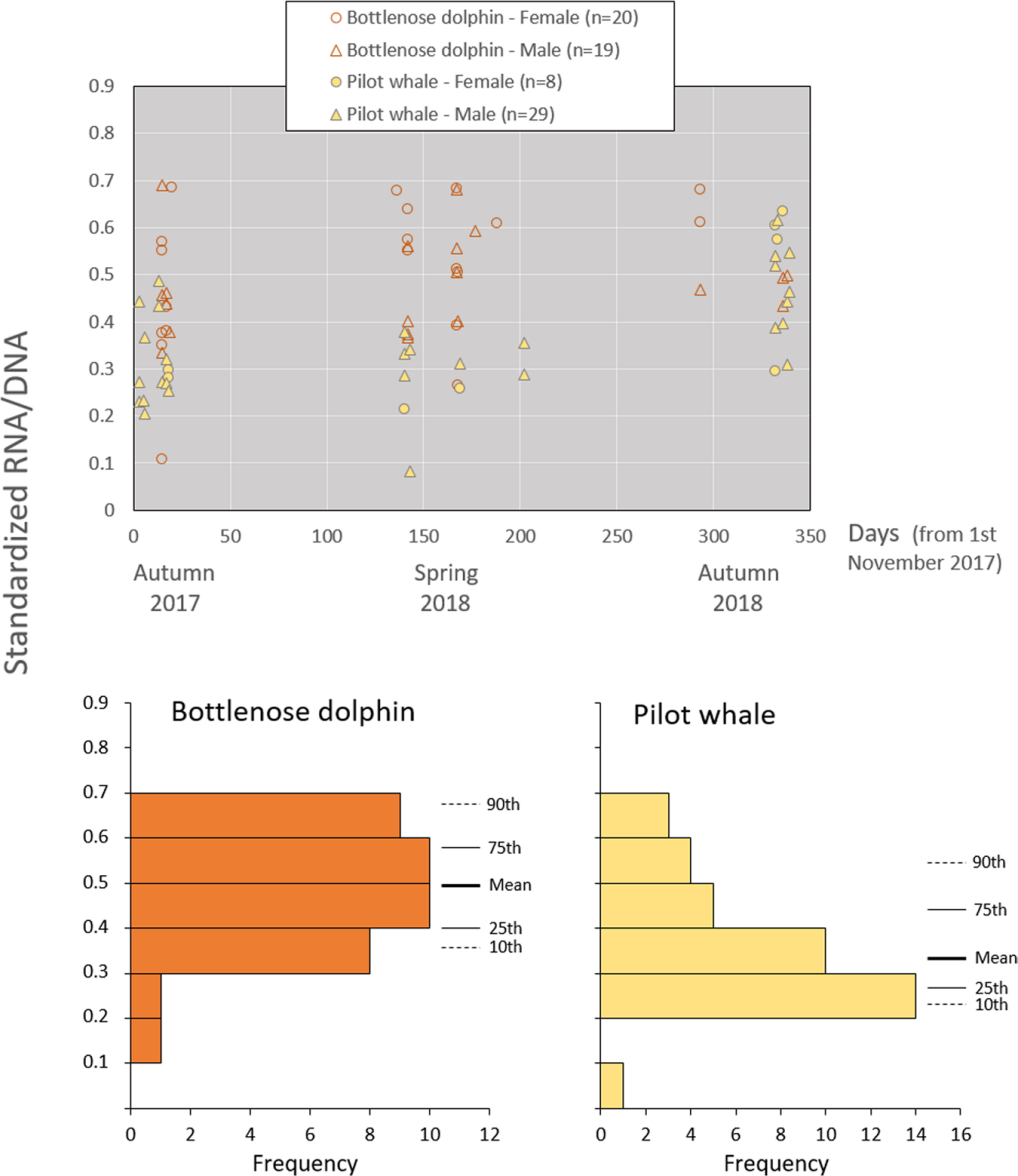
Standardized RNA/DNA ratios of common bottlenose dolphins (n = 39) and short-finned pilot whales (n = 37) per sexes throughout the study period. Frequency histograms, means and percentiles 10, 25, 75, and 90^th^ are used to illustrate a higher significant ecophysiological condition in bottlenose dolphins (p < 0.001, Table 2a); yet this difference should be interpreted with caution given that might be related to species and not to environmental conditions. The percentile approach shows that, in general, the lower percentile (10^th^) is not closer to the sample mean than the 75^th^ percentile, which suggests that the sampled populations (especially of bottlenose dolphins) contain a high number of individuals in an adequate nutritional condition.

Tissue samples were collected from free-ranging common bottlenose dolphins and short-finned pilot whales in the southern waters of Madeira Island (Portugal, Fig. 1). Madeira lies in a warm-temperate latitude, is surrounded by oceanic waters that are mainly oligotrophic, and is characterized by a narrow continental shelf, steep submarine canyons, and deep waters^38,39^. Field work comprised three dedicated campaigns covering distinct seasons: autumn 2017 (3^rd^ to 20^th^ of November), spring 2018 (16^th^ of March to 21^st^ of May), and autumn 2018 (28^th^ of September to 05^th^ of October). Exceptionally, three samples collected during late summer 2018 were included in the later bin in order to increase sample size (Fig. 2). Samples were obtained through a biopsy darting system (150-lb crossbow, with arrows and darts specially designed for small cetaceans by Finn Larsen, Ceta-Dart^40^), shot by experienced researchers carrying legal permits (see ‘Ethical approval’). Biopsy samples targeted the flanks of the animals, immediately below the dorsal fin. Samples were taken from apparently healthy adults, i.e. large and robust animals with no signs of emaciation^41,42^, and that were not carrying calves, i.e. not swimming in synchrony with young individuals. This minimized any potential bias associated with the analysis, given that the physiological condition of animals in poor health condition, calves, and lactating mothers might be different, and well as short-term stress induced by biopsying.

At sea, samples were immediately stored on liquid nitrogen. In the laboratory (LB3 of the University of Madeira), for each biopsy sample, the skin (0.5 cm in diameter) was separated from the blubber, and about ¼ of the skin tissue was separated for the determination of nucleic acids and another ¼ for the determination of sex, and stored at −80 °C. Prior to analyses, the skin tissue for the determination of nucleic acids was lyophilized for 48 hours at 60 °C and at a low pressure of about 10^−1^ atm, which was optimised for total removal of the water. Then, the skin tissue was weighed (±1 μg dry weight [DW]) on an electronic microbalance (Sartorius M5P).

Additionally, sighting data and individual identification photographs of the biopsied animals were taken. The sighting data comprised the coordinates (the closest to the individual or group), date, initial and end time, reaction to biopsy, species, presence of calves, age class, and best estimate of group size. Most parameters were determined at sea by experienced researchers and confirmed a posteriori using photographs. The collection of photographs from the cetaceans (left-and right-side of each individual) followed standard procedures^43^ using digital cameras with lenses and was simultaneous with biopsies.

### Ecophysiological condition

Ecophysiological condition was assessed using the averages RNA (µg mg^−1^ DW), DNA (µg mg^−1^ DW), and sRD (standardized RNA/DNA ratios). The concentration of these nucleic acids was quantified following the procedures described in Caldarone *et al*.^44^ and Esteves *et al*.^45^. Briefly, the skin tissues of the biopsied cetaceans were chemically (cold sarcosyl Tris-EDTA extraction buffer) and mechanically (through sonification – 3 pulses 50 A for 1 min, and vortex) homogenised, and after centrifugation the tissues were isolated. Different volumes of supernatant were placed into fluorescent plate wells with Tris buffer according to sample dry-weight. Finally, 30 µl of specific nucleic acid fluorochrome dye GelRED (GR) was added into each well for the fluorescent reading of nucleic acids. Fluorescence was measured on a microplate reader (Biotek synergy HT model SIAFRTD) using an excitation wavelength of 365 nm and an emission wavelength of 590 nm, which allowed to determine the total stable amount of DNA and RNA (mainly ribosomal) in each sample. After the first read (total fluorescence of RNA and DNA), ribonuclease A (type-II A) solution was activated by incubating the Fluorescent Plates at 37 °C for 30 minutes. The plates were read again to determine the total amount of DNA fluorescence. In each plate where the samples were analysed “only-DNA” and “only-RNA” control sample were run, and a RNAase digestion was applied to all the samples and standards to be sure that RNA digestion was complete and no DNA degradation occurred.

Finally, RNA fluorescence was calculated as the difference between total fluorescence (first scan) and the fluorescence after RNAase activation (second scan). Standard curve of DNA-GR and RNA-GR with known concentrations of λ-bacteriophage DNA (0.25 μg μl^−1^) and 16S–23S *E. coli* RNA (4 μg μl^−1^) (Roche), were created to determine nucleic acids concentrations. The ratio of DNA and RNA slopes ranged from 4.74 to 7.10. RNA/DNA ratios were standardized based on this information and the reference slope ratio of 2.4 (following Caldarone *et al*.^46^). The determination of the nucleic acids was carried out at CCMAR (see affiliations) facilities.

### Genetic sexing

Genomic DNA was extracted from the samples using a standard high-salt protocol as outlined in Sambrook *et al*.^47^. Multiplex PCR reactions aimed to amplify both ZFX and SRY gene fragments, as described in Rosel^48^, were carried out using Phusion Flash High-Fidelity PCR Master Mix (Thermo Scientific) in 20 µl reactions. The ZFX encodes a member of the krueppel C2H2-type zinc-finger protein family, while the SRY encodes a transcription factor that is a member of the high mobility group (HMG)-box family of DNA-binding proteins^49^ (Stelzer *et al*., 2016). The amplification conditions used in this study were as follows: initial denaturation for 10 seconds at 98 °C followed by 35 cycles of 1 second at 98 °C, 5 seconds at 51 °C, and a final extension of 15 seconds at 72 °C.

Due to difficulties in performing multiplex PCR reactions, we opted to perform single PCR reactions with only one set of primers (thus two PCR reactions per sample)^48,50,51^. To confirm whether the desired genes were amplified, several electrophoresis bands from different samples were sequenced. PCR products were cut from the gel, purified with the NZYGelpure (NZYTech) and sent to direct sequencing (Sanger sequencing) using the light run sequencing service of GATC Biotech (http://www.gatc-biotech.com/en/sanger-services/lightrun-sequencing.html). DNA sequences were analyzed using the BioEdit Sequence Alignment Editor version 7.0.4.1^52^ and aligned against reference sequences from GenBank. The determination of the sex was carried out at CIIMAR (see affiliations) facilities.

### Determination of residency patterns

Individual identification photographs of the biopsied pilot whales were compared with an existing long-term digital photographic-identification catalogue of this species. The catalogue comprises images collected in the same area where biopsies were taken, in the south of Madeira Island, since 2003. The catalogue was compiled by OOM (see affiliations) and most of its images were collected during whale-watching trips. The comparison was limited to pilot whales given that most bottlenose dolphins were not photographed properly during biopsying (move faster than pilot whales in general) and/or were not naturally well-marked. Of all the biopsied pilot whales, only one animal was not properly photographed (i.e. poor-quality photograph).

The compilation of the catalogue was based in Würsig and Würsig^53^, and detailed descriptions are given in Alves *et al*.^35^. Briefly, matching consisted of comparing the best processed image of each individual, based primarily on the number of unique notches on the dorsal fin, which allowed matching left-and right-side independently, and using fin shape or scars only to confirm matches^54^. Comparisons were carried out visually (e.g. Robbins *et al*.^55^), and only high-quality images (as illustrated in Alves *et al*.^34^) and matches with 100% certainty by three experienced researchers (F.A., M.W., A.D.) were used in the present study.

The establishment of residency patterns was based on the data set of individual-specific encounter histories (from 2003 to 2018). Individuals that exhibited multiyear and year-round site fidelity (photographed ≥15 times in at least three years and all four seasons; although most animals were photographed ≥63 times over 12 years) were termed residents; individuals photographed once (i.e. not previously catalogued) and not mixed with catalogued animals (to avoid erroneous classification of new/non-catalogued resident or visitor animals) were termed transients; and individuals that exhibited multi-year but seasonal specific presence (i.e. in only one or two seasons) were considered visitors (adapted from Alves *et al*.^34^). This was restricted to well-marked adult individuals in order to minimize erroneous classifications.

### Data analyses

Descriptive (arithmetic means and percentiles) and inferential statistics (analysis of variance ‘ANOVA’ tests) were used to illustrate and test for significant differences (α = 0.05) in the biochemical indices between (a) species, (b) seasons (autumn 2017, spring 2018, and autumn 2018) and sexes in bottlenose dolphins, (c) seasons and sexes in pilot whales, and (d) residency patterns (residents, transients, and visitors) in pilot whales. The small sample size of some residency pattern bins did not allow us to test for differences between sexes and seasons within this variable. Significant differences were followed by a post hoc Tukey test. The Kolmogorov-Smirnov and Levene’s tests were used to test the ANOVA assumptions (normality and homogeneity of variances, respectively). All analyses were carried out using the R 3.5.3 statistical package^56^.

## Results

Standardized RNA/DNA ratios (sRD) were obtained for 39 bottlenose dolphins (20 females and 19 males) and 37 pilot whales (8 females and 29 males) (Table 1). Ecophysiological condition index of bottlenose dolphins (mean = 0.49; percentiles 10, 25, 75, and 90^th^ = 0.36, 0.40, 0.58, and 0.68, respectively) was significantly higher than pilot whales (mean = 0.37; percentiles = 0.23, 0.27, 0.44, and 0.56, respectively) (Table 2a, Fig. 2). The RNA and DNA concentrations were not significantly different between species (p = 0.40 for RNA, and p = 0.63 for DNA; SI1 and SI2, respectively).

**Table 1:** Number of biopsies from bottlenose dolphins (n = 39) and short-finned pilot whales (n = 37) per season and sex, used to determine the biochemical indices.

Species	Bottlenose dolphin		Pilot whale	
Season\Sex	females	males	females	males
autumn 2017	8	6	2	12
spring 2018	10	9	2	8
autumn 2018	2	4	4	9
Total	20	19	8	29

**Table 2:** Results of analysis of variance (ANOVA) to test if there are significant differences in the standardized RNA/DNA ratios between (a) the two species*, (b) seasons (autumn 2017, spring 2018, and autumn 2018) and sexes in bottlenose dolphins, (c) seasons and sexes in short-finned pilot whales, and (d) residency patterns (residents, transients, and visitors) in short-finned pilot whales.

Hypothesis	ANOVA	Source	DF	F-stat	p
(a) between species*	one-way	Species	1	17.880	**<0.001**
(b) between seasons and sexes in bottlenose dolphins	two-way	Season	2	1.793	0.182
		Sex	1	0.815	0.373
		Season × Sex	2	1.182	0.319
(c.1) between seasons in pilot whales	one-way	Season	2	15.710	**<0.001**
(c.2) between sexes in pilot whales	one-way	Sex	1	0.493	0.487
(d) between residency patterns in pilot whales	one-way	Residency	2	8.865	**0.001**

DF - degrees of freedom, F-stat - F-statistic, p - significance value (in bold when <0.05). Results of the post-hoc tests for (c.1) and (d) in SI2 and SI4, respectively. *Inter-specific differences should be interpreted with caution given that might be related to species and not to environmental conditions.

**Figure 3:**
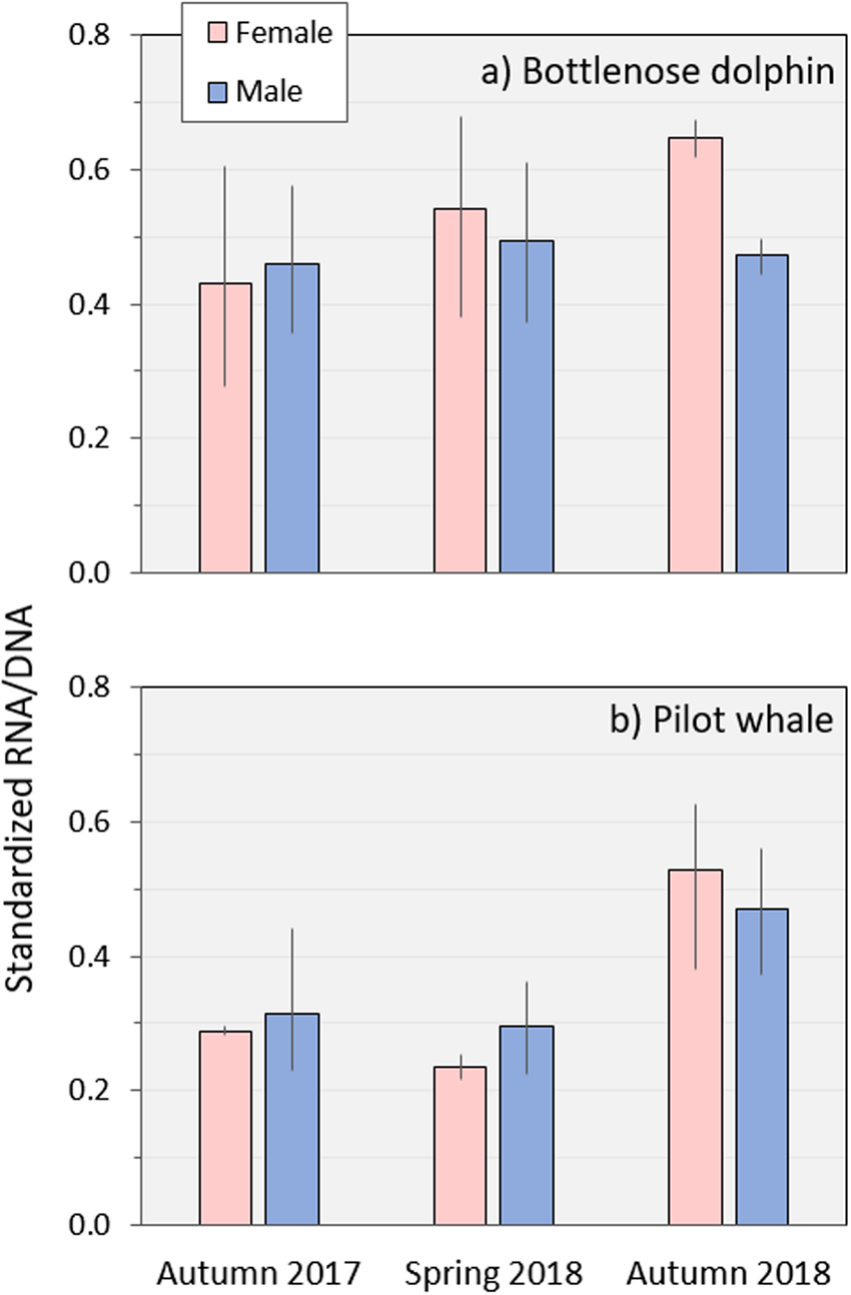
Biochemical condition (mean and percentiles 10 and 90^th^ of the standardized RNA/DNA ratios) of bottlenose dolphins (**a**) and pilot whales (**b**) per sexes and seasons. Significant higher values (p < 0.001) were found only between autumn 2018 and the remaining seasons in pilot whales (Table 2c.1, SI3). The absence of significant differences between sexes (Table 2b for bottlenose dolphins and Table 2c.2 for pilot whales) increases the applicability of using RNA/DNA ratios in large vertebrates (due to reduced sex bias).

In bottlenose dolphins, no significant changes were found in the sRD between sexes, between seasons, and neither in their interaction (Table 2b, Fig. 3). The RNA and DNA concentrations were significantly different in the factor season, with higher values in autumn 2018 (in both sexes) (SI1 and SI2). In pilot whales, the sRD was significantly different only between seasons (Table 2c.1), with higher values in autumn 2018 (in both sexes) (Figs. 3, SI3). The RNA and DNA concentrations showed the same pattern of the sRD, with significantly higher values in autumn 2018 (SI1 and SI2). The interaction of seasons and sexes could not be determined in pilot whales (i.e. one-way ANOVA tests were run independently for each factor instead of a two-way ANOVA) due to the low number of biopsied females in each season.

**Figure 4:**
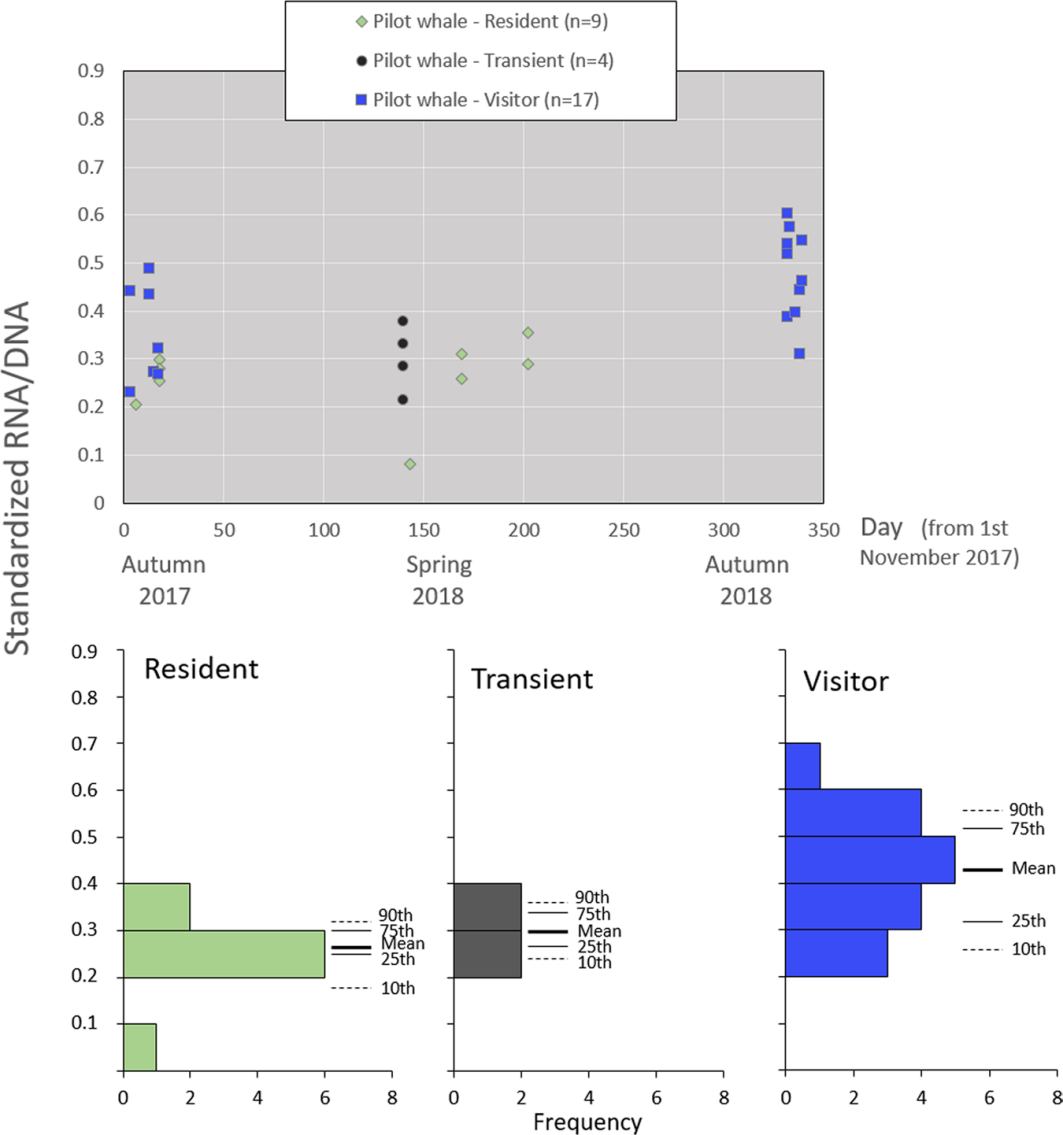
Standardized RNA/DNA ratios between pilot whales with distinct residency patterns throughout the study period. Frequency histograms, means and the percentile approach are used to illustrate a higher ecophysiological condition in visitors, which was significantly different from residents (p = 0.001, Table 2d, SI4). Based on the individual capture histories between 2003 and 2018, we classified: (i) residents, as individuals that exhibited multi-year and year-round (i.e. in the four seasons) site fidelity, (ii) transients, as individuals captured only once and not mixed with catalogued animals, and (iii) visitors, as individuals that exhibited multi-year but seasonal specific presence (i.e. in only one or two seasons); see ‘Material and methods’ for details.

Residency patterns in pilot whales were attributed to 9 residents, 4 transients, and 17 visitors. The biochemical condition (sRD) was significantly higher in visitors (mean = 0.43; percentiles 10, 25, 75, and 90^th^ = 0.27, 0.32, 0.52, and 0.56, respectively) than in residents (mean = 0.26; percentiles = 0.18, 0.25, 0.30, and 0.32, respectively) (Table 2d, Figs. 4, SI4). The RNA and DNA concentrations showed the same general pattern of the sRD (SI1 and SI2).

## Discussion

In this study, we used a percentile approach to obtain a better representation of the animal’s relative ecophysiological condition^57^. Because in field populations the condition is stochastically distributed, population sample percentiles can then describe the shape of the underlying distribution and the inter-individual variability, which facilitate the interpretation of what might be considered a poor or good condition^57,58^. In our case, and although with inter- and intra-specific differences (as discussed below), in general, the sample contains individuals in good condition given that the lower percentile (the 10^th^ of RNA/DNA ratios - indicator of the lowest life-stage-specific condition^57,59^) is not close to the mean. Moreover, the 75^th^ percentile is closer to the sample mean than the 10^th^ percentile, which suggests an adequate health status. Hence, such relative nutritional condition of these highly mobile top predators indicate that the marine biogeographic region of Macaronesia may be considered an adequate habitat for those species, but that (especially in resident pilot whales) future population and ecosystem monitoring should be carried out (e.g. survival rates, PCB’s, heavy metals). Information on the ecophysiological condition of marine megafauna is scarce and mainly correlated with growth, and in the case of highly mobile predators such as cetaceans, most health indices have derived from photogrammetry of body condition or stress hormones^60^. In this study, and although preliminary, we demonstrate the use of biochemical indices as an innovative way to infer on the ecophysiological traits of bottlenose dolphins and pilot whales, and that these indices can provide an overall picture of ecosystem health.

Our specific goals explored differences in the ecophysiological condition at inter- and intra-specific levels, from which three main findings have emerged. These were based on the principle that individuals in good nutritional condition generally have high levels of RNA/DNA ratios, whereas individuals from related phylogenetic groups undergoing dietary restriction have a lower amount of RNA in their cells and hence a lower rate of RNA/DNA ratios^1,3,61^, given that the amount of RNA directly involved in protein synthesis varies with age, disease-state or environmental conditions^62,63^. Our first main finding is that RNA/DNA ratios varied significantly in animals with different residency patterns. RNA/DNA ratios were significantly higher in autumn 2018 in pilot whales, and significantly higher in visitors (when compared to resident pilot whales). But curiously, a closer inspection revealed that all pilot whales with a known residency pattern sampled in autumn 2018 were identified as visitors (Fig. 4). This suggests that the higher values obtained in autumn 2018 could correspond to visitors. This is supported by the higher RNA/DNA ratios from visitors (when compared to residents) in autumn 2017, and by the absence of significant differences in RNA/DNA ratios in bottlenose dolphins between seasons. Indeed, visitor pilot whales have different capture probabilities (when compared to those from residents and transients) in Madeira^30,35^, which should reflect a distinct biogeographic ecology for this ecotype, i.e. with different spatial structure, movements and/or feeding habitats. The higher RNA/DNA ratios thus suggest that visitor pilot whales are in better nutritional condition and are likely to be more robust or ‘adapted’ to a wider spectrum of pelagic habitats. Although different ecotypes displaying different feeding habits have been described in other cetacean species such as in killer whales (*Orcinus orca*) (e.g. Foote *et al*.^64^), there is no available information on their ecophysiological condition to support our findings. Therefore, the suggestion presented here should be viewed as a hypothesis to be explored in future research. This could include, for example, the assessment of the ecophysiological condition (based on the approached presented here), or the study of the nutritional ecology based on a geometry framework that distinguishes specific nutrients and calories^65^, in distinct ecotypes.

Second, no significant differences were obtained between sexes in any of the species. Chícharo *et al*.^66^ found higher RNA/DNA ratios in females than in males in fishes, crustaceans, and bivalves, suggesting that sexual dimorphism in addition to physiological and behavioural differences may have accounted for those differences. Therefore, those authors mentioned that the effect of sex should be taken into account on studies using nucleic acid concentrations. In this study, we used genetic sexing due to the difficulties in determining the sex of bottlenose dolphins and pilot whales at sea^21,67^, in order to comprise the effect of sex in our analyses. Taking into consideration that mammals lactate, which could imply different RNA for females, and that calves of both species have been commonly recorded in Madeiran waters^27,68^, higher variability in the RNA/DNA ratios could be expected in females, in at least one of the seasons. However, our findings showed similar ratios between sexes, which is in agreement with a study on smooth dogfish sharks (*Mustelus canis*)^69^. This suggests that, contrarily to invertebrates and small fishes, the RNA directly involved in protein synthesis does not vary significantly between females and males in large top predators; yet this lacks support from further studies. The fact that females and males do not provide different bias to the analysis could be a major advantage and applicability for the use of RNA/DNA ratios in large vertebrates.

Third, bottlenose dolphins showed significantly higher RNA/DNA ratios than pilot whales. However, and even if it is tempting to mention that this could be related to environmental conditions or reflect biological differences between the two species given all animals were biopsied close in time and space using the same *in situ* and laboratory methodological procedures, in fact we did not consider species-specific amounts of RNA and DNA and thus any differences might be related to species. Therefore, assuming a better nutritional condition in bottlenose dolphins (based for example on its more diversified diet^20,21,70–72^ or possible higher resilience to human-induced activities) should be interpreted with caution. In the future, such inter-specific discrepancy could be better interpreted if RNA/DNA ratios became available for other populations and/or species.

As an overview, this study has shown that RNA/DNA ratios can provide important information on the physiological and nutritional status of highly mobile large predators, supporting the use of these biochemical indices as indicators of the health status of marine organisms and ecosystems. Nevertheless, the application of this methodology on such animals offers limitations. First, obtaining tissue samples from free-ranging animals requires experienced researchers, and is expensive and time-consuming for most species; not to mention that many of these target taxa are endangered and/or not hunted commercially. Second, RNA/DNA ratios have been successfully used as important biomarkers of growth rates^61,73^, yet combining biochemical analyses with morphometric measurements is challenging in wild fast-moving megafauna due to difficulties in obtaining precise body lengths^74^. The same may apply to age class or sex determination (known only *a posteriori* in our case) of the sampled animal, which can be useful variables to take into account in data analysis. Additionally, different tissues or body parts can have different RNA or DNA-tissue relationships^75^, so caution should be made to obtain tissue samples from the same body part, as we did, in order to minimize potential bias. Finally, a comparison between studies or species requires similar metabolic responses and methodologies. Overall, the combination of methodologies used in this study (comprising biochemical analyses to determine the nucleic acid-derived indices, genetic analyses to determine sex, and photographic-identification to establish residency patterns), allowed a more comprehensive analysis of the ecophysiological condition in highly mobile large predators and in providing an ecological framework. Although this study constitutes a first approach in the use of nucleic acid derived indices in these animals, it deserves further attention to be used in other top predators and ecosystems.

### Ethical approval

Biopsies were obtained in accordance with the relevant guidelines and regulations imposed by Instituto de Florestas e Conservação da Natureza (Instituto Português - Região Autónoma da Madeira) and under sampling permits 1.856/2017, 508/2018 and 10661/2018 from the same Portuguese institute.

## Supplementary information

Supplementary Information2.

Supplementary Information3.

Supplementary Information4.

Supplementary Information5.

Supplementary Information.
