# Supplementary material for: Ecophysiological traits of highly mobile large marine predators inferred from nucleic acid derived indices
Source: Sci Rep. 2020 Mar 16;10:4752. doi: 10.1038/s41598-020-61769-7 (PMC7075925; doi:10.1038/s41598-020-61769-7)
Supplement: Supplementary file 1 — Supplementary Information2. [file 41598_2020_61769_MOESM1_ESM.pdf]

Supplementary information

Ecophysiological traits of highly mobile large marine predators inferred from nucleic acid derived indices

F. Alves, M. Dromby, V. Baptista, R. Ferreira, A. M. Correia, M. Weyn, R. Valente, E. Froufe, M. Rosso, I. Sousa-Pinto, A. Dinis, E. Dias & M. A. Teodósio

SI1. Statistics for the average RNA concentrations.

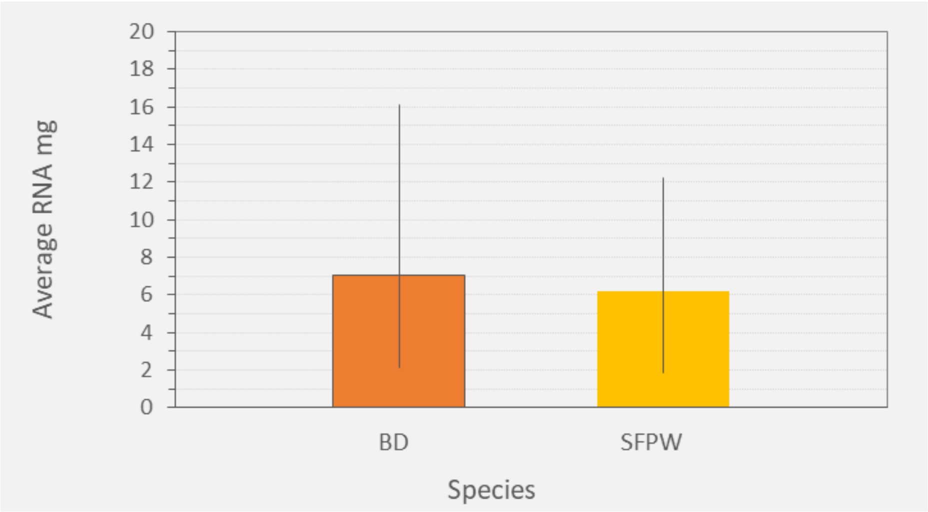

Figure SI1.1. Means and percentiles 10 and 90th of the average RNA concentrations (µg mg^{-1} dry weight) for common bottlenose dolphins (BD, n=39) and short-finned pilot whales (SFPW, n=37).

In the common bottlenose dolphin, the mean = 7.08, and the percentiles 10, 25, 75, and 90th = 2.14, 3.43, 8.48, and 16.09, respectively. In the short-finned pilot whale, the mean = 6.16, and the percentiles 10, 25, 75, and 90th = 1.83, 2.93, 7.28, and 12.21, respectively.

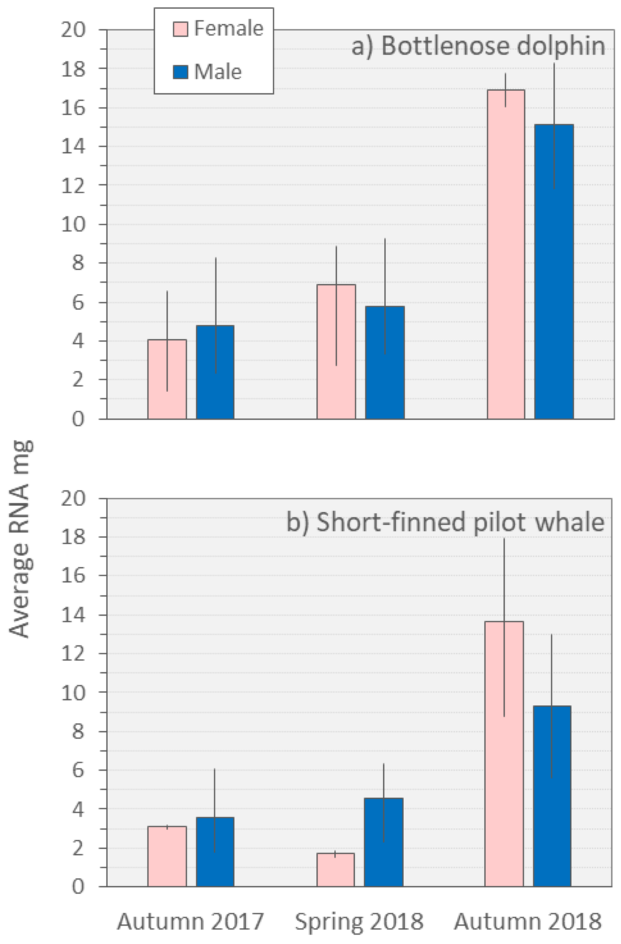

Figure SI1.2. Means and percentiles 10 and 90th of the average RNA concentrations (µg mg^{-1} dry weight) for bottlenose dolphins (a) and pilot whales (b) per sexes and seasons.

Table SI1.1. Results of analysis of variance (ANOVA) to test if there are significant differences in the RNA concentrations between a) the two species, b) seasons (autumn 2017, spring 2018, and autumn 2018) and sexes in bottlenose dolphins, c) seasons and sexes in short-finned pilot whales, and d) residency patterns (residents, transients, and visitors) in short-finned pilot whales. DF - degrees of freedom, F-stat - F-statistic, p - significance value (in bold when <0.05).

Hypothesis	ANOVA	Source	DF	F-stat	p
a) between species	one-way	Species	1	0.715	0.401
b) between seasons and sexes in bottlenose dolphins	two-way	Season	2	26.437	**<0.001**
		Sex	1	0.277	0.602
		Season X Sex	2	0.443	0.646
c.1) between seasons in pilot whales	one-way	Season	2	23.020	**<0.001**
c.2) between sexes in pilot whales	one-way	Sex	1	1.834	0.184
d) between residency patterns in pilot whales	one-way	Residency	2	4.154	**0.027**

Table SI1.2. Means and percentiles of the average RNA concentrations (μg mg^{-1} dry weight) per residency pattern in short-finned pilot whales.

Pilot whales	Mean	Percentil 10th	Percentil 25th	Percentil 75th	Percentil 90th	Number of samples
Residents	3.28	1.64	1.89	3.23	6.03	9
Trasients	4.39	2.38	3.68	5.77	5.86	4
Visitors	7.55	3.03	4.51	10.95	13.57	17
