# Supplementary material for: Ecophysiological traits of highly mobile large marine predators inferred from nucleic acid derived indices
Source: Sci Rep. 2020 Mar 16;10:4752. doi: 10.1038/s41598-020-61769-7 (PMC7075925; doi:10.1038/s41598-020-61769-7)
Supplement: Supplementary file 3 — Supplementary Information4. [file 41598_2020_61769_MOESM3_ESM.pdf]

Supplementary information

Ecophysiological traits of highly mobile large marine predators inferred from nucleic acid derived indices

F. Alves, M. Dromby, V. Baptista, R. Ferreira, A. M. Correia, M. Weyn, R. Valente, E. Froufe, M. Rosso, I. Sousa-Pinto, A. Dinis, E. Dias & M. A. Teodósio

SI3. Outputs of the one-way ANOVA and post-hoc Tukey test for the standardized RNA/DNA ratios between seasons (Aut17 - autumn 2017, Spr18 - spring 2018, Aut18 - autumn 2018) in short-fined pilot whales.

```
> AnovaModel.5 <- aov(sRD ~ SeasonYear, data=Dataset)
> summary(AnovaModel.5)
             Df Sum Sq Mean Sq F value    Pr(>F)
SeasonYear   2 0.2975 0.14874   15.71 0.0000148 ***
Residuals    34 0.3220 0.00947
---
Signif. codes:  0 '***' 0.001 '**' 0.01 '*' 0.05 '.' 0.1 ' ' 1

> with(Dataset, numSummary(sRD, groups=SeasonYear, statistics=c("mean",
+   "sd")))
           mean         sd     data:n
Aut17 0.3117764 0.08750118     14
Aut18 0.4868477 0.11395191     13
Spr18 0.2844737 0.08605875     10

> local({
+   .Pairs <- glht(AnovaModel.5, linfct = mcp(SeasonYear = "Tukey"))
+   print(summary(.Pairs)) # pairwise tests
+   print(confint(.Pairs)) # confidence intervals
+   print(cld(.Pairs)) # compact letter display
+   old.oma <- par(oma=c(0,5,0,0))
+   plot(confint(.Pairs))
+ })
```

Simultaneous Tests for General Linear Hypotheses

Multiple Comparisons of Means: Tukey Contrasts

Fit: aov(formula = sRD ~ SeasonYear, data = Dataset)

```
Linear Hypotheses:
                     Estimate Std. Error t value Pr(>|t|)
Aut18 - Aut17 == 0  0.17507    0.03748   4.671   <0.001 ***
Spr18 - Aut17 == 0 -0.02730    0.04029  -0.678    0.778
Spr18 - Aut18 == 0 -0.20237    0.04093  -4.944   <0.001 ***
---
Signif. codes:  0 '***' 0.001 '**' 0.01 '*' 0.05 '.' 0.1 ' ' 1
(Adjusted p values reported -- single-step method)
```

Simultaneous Confidence Intervals
Multiple Comparisons of Means: Tukey Contrasts
Fit: aov(formula = sRD ~ SeasonYear, data = Dataset)

```
Quantile = 2.4496
95% family-wise confidence level
Linear Hypotheses:
                    Estimate   lwr      upr
Aut18 - Aut17 == 0  0.17507  0.08325  0.26689
Spr18 - Aut17 == 0 -0.02730 -0.12600  0.07140
Spr18 - Aut18 == 0 -0.20237 -0.30265 -0.10210
```

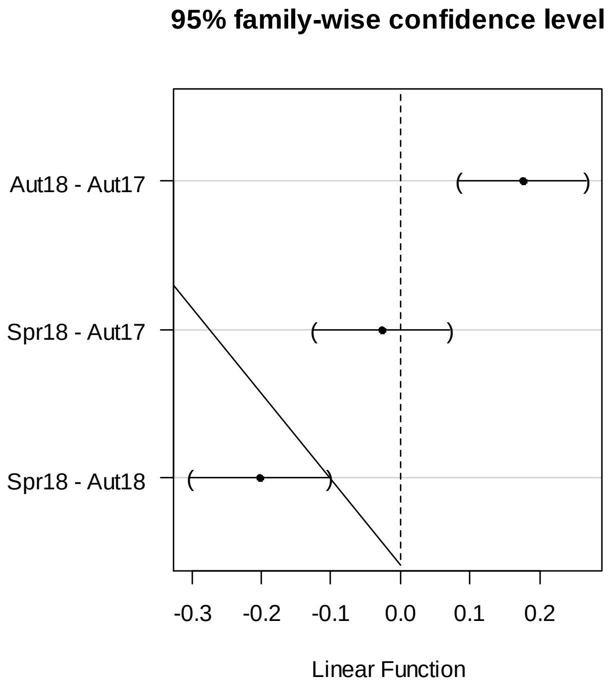
95% family-wise confidence level
Aut18 - Aut17
Spr18 - Aut17
Spr18 - Aut18
-0.3
-0.2
-0.1
0.0
0.1
0.2
Linear Function
