# Supplementary material for: Ecophysiological traits of highly mobile large marine predators inferred from nucleic acid derived indices
Source: Sci Rep. 2020 Mar 16;10:4752. doi: 10.1038/s41598-020-61769-7 (PMC7075925; doi:10.1038/s41598-020-61769-7)
Supplement: Supplementary file 4 — Supplementary Information5. [file 41598_2020_61769_MOESM4_ESM.pdf]

Supplementary information

Ecophysiological traits of highly mobile large marine predators inferred from nucleic acid derived indices

F. Alves, M. Dromby, V. Baptista, R. Ferreira, A. M. Correia, M. Weyn, R. Valente, E. Froufe, M. Rosso, I. Sousa-Pinto, A. Dinis, E. Dias & M. A. Teodósio

SI4. Outputs of the one-way ANOVA and post-hoc Tukey test for the standardized RNA/DNA ratios between residency patterns (residents, transients, and visitors) in short-finned pilot whales.

```
> AnovaModel.7 <- aov(sRD ~ ResidencyPattern, data=Dataset)
> summary(AnovaModel.7)
                  Df Sum Sq Mean Sq F value Pr(>F)
ResidencyPattern  2 0.1788 0.08942   8.865    0.0011 **
Residuals        27 0.2723 0.01009
---
Signif. codes:  0 '***' 0.001 '**' 0.01 '*' 0.05 '.' 0.1 ' ' 1
7 observations deleted due to missingness

> with(Dataset, numSummary(sRD, groups=ResidencyPattern, statistics=c("mean",
+   "sd")))
              mean         sd data:n
Resident  0.2590072 0.07818238      9
Transient 0.3021255 0.07010728      4
Visitor   0.4258247 0.11420728     17

> local({
+   .Pairs <- glht(AnovaModel.7, linfct = mcp(ResidencyPattern = "Tukey"))
+   print(summary(.Pairs)) # pairwise tests
+   print(confint(.Pairs)) # confidence intervals
+   print(cld(.Pairs)) # compact letter display
+   old.oma <- par(oma=c(0,5,0,0))
+   plot(confint(.Pairs))
+ })
```

```
Simultaneous Tests for General Linear Hypotheses

Multiple Comparisons of Means: Tukey Contrasts


Fit: aov(formula = sRD ~ ResidencyPattern, data = Dataset)

Linear Hypotheses:
                            Estimate Std. Error t value Pr(>|t|)
Transient - Resident == 0  0.04312    0.06035   0.714  0.75336
Visitor - Resident == 0    0.16682    0.04140   4.029  0.00108 **
Visitor - Transient == 0   0.12370    0.05581   2.216  0.08395 .
---
Signif. codes:  0 '***' 0.001 '**' 0.01 '*' 0.05 '.' 0.1 ' ' 1
(Adjusted p values reported -- single-step method)


Simultaneous Confidence Intervals
Multiple Comparisons of Means: Tukey Contrasts
Fit: aov(formula = sRD ~ ResidencyPattern, data = Dataset)

Quantile = 2.4663
95% family-wise confidence level


Linear Hypotheses:
                            Estimate lwr      upr
Transient - Resident == 0  0.04312 -0.10573  0.19196
Visitor - Resident == 0    0.16682  0.06471  0.26893
Visitor - Transient == 0   0.12370 -0.01395  0.26135
```

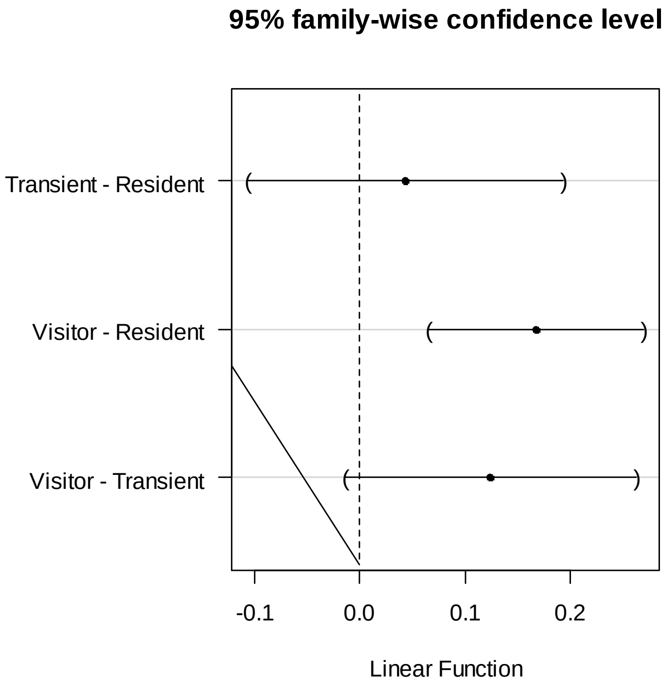
95% family-wise confidence level
Transient - Resident
Visitor - Resident
Visitor - Transient
-0.1
0.0
0.1
0.2
Linear Function
